# Anti-Inflammatory and Mineralization Effects of an ASP/PLGA-ASP/ACP/PLLA-PLGA Composite Membrane as a Dental Pulp Capping Agent

**DOI:** 10.3390/jfb13030106

**Published:** 2022-07-29

**Authors:** Wenjuan Yan, Fenghe Yang, Zhongning Liu, Quan Wen, Yike Gao, Xufeng Niu, Yuming Zhao

**Affiliations:** 1 Department of Pediatric Dentistry, Peking University School and Hospital of Stomatology & National Center of Stomatology & National Clinical Research Center for Oral Diseases & National Engineering Research Center of Oral Biomaterials and Digital Medical Devices & Beijing Key Laboratory of Digital Stomatology & Research Center of Engineering and Technology for Computerized Dentistry Ministry of Health & NMPK Key Laboratory for Dental Materials, Beijing 100081, China; yanwenjuan85@163.com (W.Y.); gaoyike@pku.edu.cn (Y.G.); 2 Department of First Clinical Division, Peking University School and Hospital of Stomatology & National Center of Stomatology & National Clinical Research Center for Oral Diseases & National Engineering Research Center of Oral Biomaterials and Digital Medical Devices & Beijing Key Laboratory of Digital Stomatology & Research Center of Engineering and Technology for Computerized Dentistry Ministry of Health & NMPK Key Laboratory for Dental Materials, Beijing 100081, China; wincky.ls@163.com; 3 Key Laboratory of Biomechanics and Mechanobiology, Ministry of Education, Beijing Advanced Innovation Center for Biomedical Engineering, School of Biological Science and Medical Engineering, Beihang University, Beijing 100083, China; yfh1993@buaa.edu.cn; 4 Department of Prosthodontics, Peking University School and Hospital of Stomatology & National Center of Stomatology & National Clinical Research Center for Oral Diseases & National Engineering Research Center of Oral Biomaterials and Digital Medical Devices & Beijing Key Laboratory of Digital Stomatology & Research Center of Engineering and Technology for Computerized Dentistry Ministry of Health& NMPK Key Laboratory for Dental Materials, Beijing 100081, China; lzn45@163.com

**Keywords:** aspirin, anti-inflammation, amorphous calcium phosphate composite scaffold, dental pulp-capping material, dental pulp stem cell

## Abstract

Dental pulp is essential for the development and long-term preservation of teeth. Dental trauma and caries often lead to pulp inflammation. Vital pulp therapy using dental pulp-capping materials is an approach to preserving the vitality of injured dental pulp. Most pulp-capping materials used in clinics have good biocompatibility to promote mineralization, but their anti-inflammatory effect is weak. Therefore, the failure rate will increase when dental pulp inflammation is severe. The present study developed an amorphous calcium phosphate/poly (L-lactic acid)-poly (lactic-co-glycolic acid) membrane compounded with aspirin (hereafter known as ASP/PLGA-ASP/ACP/PLLA-PLGA). The composite membrane, used as a pulp-capping material, effectively achieved the rapid release of high concentrations of the anti-inflammatory drug aspirin during the early stages as well as the long-term release of low concentrations of aspirin and calcium/phosphorus ions during the later stages, which could repair inflamed dental pulp and promote mineralization. Meanwhile, the composite membrane promoted the proliferation of inflamed dental pulp stem cells, downregulated the expression of inflammatory markers, upregulated the expression of mineralization-related markers, and induced the formation of stronger reparative dentin in the rat pulpitis model. These findings indicate that this material may be suitable for use as a pulp-capping material in clinical applications.

## 1. Introduction

The preservation of vital dental pulp is essential for maintaining tooth sensation, nutrition, immunity, and dentin formation; these aspects contribute to long-term prognoses [1,2]. Therefore, vital dental pulp preservation is an important goal of modern minimally invasive dentistry [3]. Currently, the most common clinical pulp-capping agent material is mineral trioxide aggregate (MTA), regarded as the clinical standard for pulp capping [4]. MTA has an excellent effect on the promotion of the formation of reparative dentin; however, its anti-inflammation effect was relatively weak [5]. Therefore, the success rate of vital pulp preservation is relatively low in the presence of inflammation [6,7]. Investigations of novel pulp-capping materials with anti-inflammatory effects could improve the success rate of vital pulp preservation and expand its indications, which have important clinical significance and practical value.

Ideal pulp-capping material for early-stage pulpitis should rapidly inhibit inflammation to create a beneficial microenvironment for dental pulp repair, after which it should promote long-term mineralization to form a hard tissue barrier [8,9,10]. In order to fulfill these requirements, there is a need for multilayer biomaterials with sequential release properties. Aspirin (ASP) is a nonsteroidal anti-inflammatory drug widely used in clinics. Its main functions include analgesia, antipyretic and anti-inflammatory; in addition, it has properties that affect the immune response and exert anti-infection and anti-biofilm activity [11]. Aspirin can reduce the expression levels of inflammatory factors in dental pulp stem cells [12], which indicates that aspirin could play an effective role in controlling pulp inflammation. Amorphous calcium phosphate (ACP) has been demonstrated to have good biodegradability and bioactivity and no cytotoxicity; ACP scaffolds have been shown to continuously release calcium and phosphorus ions. It has been widely applied in the biomedical field due to its excellent bioactivity, high cell adhesion, and adjustable biodegradation rate [13,14,15,16]. The biodegradable materials poly (L-lactic acid) (PLLA) and poly (lactic-co-glycolic acid) (PLGA) can achieve the controlled release or sustained release of drugs by adjusting the degradation rate [17,18].

The dental pulp-capping material was designed as a three-layer composite membrane. The lower layer was an ASP/PLGA membrane that contained high concentrations of ASP; the middle layer was an ASP/ACP/PLLA membrane that contained low concentrations of ASP and ACP, and the outer layer was a PLGA membrane. The lower ASP/PLGA membrane can rapidly release aspirin to control pulp inflammation; the middle ASP/ACP/PLLA membrane can perform the long-term release of calcium/phosphorus ions and aspirin to promote the formation of reparative dentin; and the outer PLGA membrane layer improves the superficial hydrophilicity of the composite membrane, which is beneficial to improving the sealing property of the pulp-capping material.

Pulpitis is inflammation caused by bacteria or toxins invading the dental pulp in the center of the tooth. Gram-negative bacteria are common microorganisms that cause pulp inflammation and necrosis. Lipopolysaccharide (LPS) is the main molecular component of these bacterial cell walls [19]. In vivo, it can activate monocyte macrophages, endothelial cells, and epithelial cells, through the cell signal transduction system, cause the synthesis and release of various cytokines and inflammatory mediators, and then initiate a series of reactions in the body. It is often used to induce inflammatory models [20]. This study aimed to evaluate the effect of the composite membrane on the proliferation, migration, and differentiation of dental pulp cells under an LPS-induced inflammation condition in vitro, as well as its potential role in the formation of reparative dentin in a rat pulpitis model in vivo, so as to provide support for its future clinical application.

## 2. Materials and Methods

### 2.1. Preparation of the ASP/ACP/PLLA Electrospun Membrane

The membrane was fabricated by using a multilayering electrospinning method. Briefly, a PLLA polymer solution (50% *w*/*v*) with aspirin at 0.12 wt% PLLA and ACP powders at 10 wt% PLLA were prepared in a 3:1 DCM: DMF solvent mixture. The solution was stirred for 1 h to obtain a uniform suspension, after which this suspension was loaded into a 20 mL syringe and electrospun at 20 kV under a steady flow rate of 1.2 mL/h. The distance between the collection foil and the syringe needle was set as 10 cm. After electrospinning for 2.5 h, the resulting ASP/ACP/PLLA membrane was separated from the collection foil and dried in a desiccator overnight. The dried ASP/ACP/PLLA membrane was cut into small pieces of equal weight and put on the collection foil for further electrospinning.

### 2.2. Preparation of the ASP/PLGA-ASP/ACP/PLLA-PLGA Electrospun Composite Membrane

A polymer solution containing 30% *w*/*v* PLGA with aspirin at 1.2 wt% PLGA was prepared in a 3:1 DCM: DMF solvent mixture. The solution was electrospun on one side of the small pieces of the ASP/ACP/PLLA membrane we prepared before at 20 kV under a steady flow rate of 1.6 mL/h with a distance of 10 cm for 0.5 h to obtain the ASP/PLGA-ASP/ACP/PLLA electrospun membrane. The other side of the ASP/ACP/PLLA membrane was electrospun for 0.5 h using a pure PLGA solution under the same conditions to obtain the final ASP/PLGA-ASP/ACP/PLLA-PLGA membrane. The resulting multilayer membranes were collected and dried in a desiccator overnight.

### 2.3. Characterization of the Composite Membrane

The membranes were mounted on metal stubs and coated with platinum for microscopical observation. Both the surface and cross-section morphologies were observed using a scanning electron microscope (SEM, FEI Quanta 250 FEG, Hillsboro, OR, USA). The diameter distribution of the nanofibers was analyzed using the image analysis program ImageJ. An energy-dispersive spectrometer (EDS) was used to determine the chemical composition of the electrospun membrane. The contact angle of the ASP/ACP/PLLA and ASP/PLGA-ASP/ACP/PLLA-PLGA membranes was measured by a contact angle measurement system (JC2000FM, POWER EACH, Shanghai, China) with a water droplet size of 0.5 μL. Moreover, transmission electron microscope (TEM) and X-ray diffraction (XRD) were employed here to identify that the ACP we loaded is in amorphous phases. For the TEM (JEOLJEM-2100, JEOL Ltd., Akishima-shi, Japan) observation, the samples were ultrasonicated in alcohol for 30 min and picked up with copper net films to observe their surface morphologies. Meanwhile, the XRD patterns were obtained from a Rigaku D/Max X-ray diffractometer (Rigaku Corporation, Tokyo, Japan) with Cu Kα radiation at 40 kV, 120 mA, and a scanning rate of 10°/min from 3° to 70°.

### 2.4. In Vitro Degradation Experiments

In vitro degradation experiments were conducted by monitoring the media pH and mass loss of the electrospun membrane during the course of 21 days. Briefly, 20 mg of the ASP/PLGA-ASP/ACP/PLLA-PLGA membrane were immersed in 3 mL of phosphate buffered solution (PBS, 0.1 M, pH 7.4) and kept at 37 °C incubator shaker. The PBS media were changed every three days with fresh PBS. The replaced PBS was collected, and the pH value of this degradation media was measured by using a pH meter (Leici PHS-3C, Shangai INESA Scientific Instrument Co., Ltd., Shanghai, China). The mass loss of the composite membrane was examined on 3, 7, 14 and 21 days using an electronic balance, and the membrane was lyophilized for 24 h before measuring.

### 2.5. In Vitro Release Experiments

In vitro release experiments were carried out to analyze the ion release (Ca^2+^, PO_4_^3−^) and aspirin release from the membrane over a period of 21 days. Before the release experiments, an acceleration study was firstly carried out according to our previous work to calculate the aspirin loading efficiency in the membrane. In order to study the release in vitro, 20 mg of the ASP/PLGA-ASP/ACP/PLLA-PLGA membrane were placed in test tubes with 3 mL of physiological saline (for Ca^2+^, PO_4_^3−^ measurement) or PBS (for aspirin measurement) and kept in an incubator shaker that is maintained at 37 °C. At predetermined time intervals, the release media in test tubes were collected and replaced with an equal amount of fresh media. The Ca^2+^, PO_4_^3−^, and aspirin concentrations were tested by a calcium colorimetric assay kit (Sigma-Aldrich, Saint Louis, MO, USA) and a phosphate ion colorimetric assay kit (Sigma-Aldrich, Saint Louis, MO, USA), as well as a multimode microplate reader (Varioskan Flash, Thermo Fisher Scientific, Waltham, MA, USA) at 278 nm/298 nm, respectively. 

### 2.6. Cell Culture

Dental pulp stem cells (DPCs) were provided by the Oral Stem Cell Bank (Beijing, China). The DPCs were cultured in an α-modified minimum essential medium (α-MEM; Gibco, St. Louis, MO, USA) with 10% fetal bovine serum (Gibco, St. Louis, MO, USA) and 100 U/mL penicillin-streptomycin (Sigma-Aldrich, St. Louis, MO, USA) at 37 °C in a 5% CO_2_ incubator. The medium was changed every 3 days. Cells were used in passages 3–5 for all of the experiments.

### 2.7. Cell Proliferation Assay 

The effect of the composite membrane on DPCs proliferation was assessed by using a Cell Counting Kit-8 (CCK-8) assay (Dojindo, Kumamoto, Japan). DPCs were seeded at a density of 3 × 103 cells/well in 96-well plates (n = 3 wells per group), cultured, and treated with the culture medium containing ASP/PLGA-ASP/ACP/PLLA-PLGA composite membrane extract, while the α-MEM was used for the control group. At 1, 3, 5, and 7 days, 10 µL of the CCK-8 solution was added to each well. The absorbance was measured at 450 nm by using an Absorbance Microplate Reader (BioTek Instruments, Winooski, VT, USA). The DPCs were treated with 1 μg/mL of lipopolysaccharide (LPS) to establish a model of inflamed dental pulp stem cells (iDPCs). The cells were treated separately with ASP, ACP/PLLA-PLGA scaffold extract, PLGA-PLLA-PLGA scaffold extract, or ASP/PLGA-ASP/ACP/PLLA-PLGA composite membrane extract; the α-MEM was used for the control group. At 1, 3, 5, and 7 days, CCK-8 assays were conducted as described previously.

### 2.8. Cell Adhesion and Migration 

Cell adhesion was observed by using phalloidin/4,6-diamino-2-phenylindole (DAPI; Invitrogen, Carlsbad, CA, USA) staining. DPCs were seeded at a density of 1 × 104 cells/well in a 24-well plate with a composite membrane or a glass slide at the bottom and cultured in the α-MEM. After 24 h, the cells were washed and fixed with paraformaldehyde. Phalloidin staining was performed for 30 min, and the nuclei were stained with DAPI for 10 min. After the cells had been washed with phosphate-buffered saline, cell skeletons were observed by using a confocal microscope (Zeiss LSM 7 Duo, Jena, Germany). The cell scratch test was used to examine the impact of each membrane treatment on DPCs migration. DPCs were seeded at 1 × 105 cells/well in a 6-well plate. When the cells reached 90% confluence, a clear and straight scratch was drawn by using a 1 mL pipette tip on the bottom of the plate; the scratch was measured by using a ruler. Exfoliated cells were washed away using phosphate-buffered saline, and the remaining DPCs were treated with the membrane extract liquid and α-MEM. The initial position and width of the scratch were recorded by photography using an inverted microscope. After culturing at 37 °C with 5% CO_2_ in a humidified incubator for 12 h, photographs were collected by using the inverted microscope at the same observation point to record the distance of cell migration; the distance was measured and analyzed by using ImageJ software.

### 2.9. Alizarin Red Staining (ARS)

ARS was performed to evaluate intracellular mineral deposition. DPCs were seeded at 6 × 104 cells/well in a 12-well plate and were then treated with LPS. Subsequently, they were cultured with ASP, ACP/PLLA-PLGA membrane extract, or ASP/PLGA-ASP/ACP/PLLA-PLGA composite membrane extract, α-MEM only was used for the control group. When the cells reached approximately 80% confluence, the medium was replaced with osteogenic differentiation media that contained 100 nM dexamethasone, 10 mM sodium β-glycerophosphate, and 10 nM L-ascorbic acid (Sigma-Aldrich, Saint Louis, MO, USA). After being cultured for 14 days, the cells were then fixed in 4% paraformaldehyde, washed with phosphate-buffered saline to remove fixative residues, and stained using a 2% ARS solution (Sigma-Aldrich, Saint Louis, MO, USA). The samples were subjected to a microscopy analysis for the qualitative evaluation of the differentiation. For the quantitative evaluation, ARS was dissolved in 5% perchloric acid; cellular absorbance was then measured at 490 nm using a Varioskan Flash (Thermo Fisher Scientific, Waltham, MA, USA) microplate spectrophotometer.

### 2.10. Quantitative Polymerase Chain Reaction (qPCR)

DPCs were seeded at 1 × 105 cells/well in a 6-well plate and were then treated with LPS. Cells were cultured for 7 days in osteogenic media with the membrane extract. The relative expression levels of osteogenic/odontogenic markers alkaline phosphatase (ALP), dentin sialophosphoprotein (DSPP), dentin matrix protein1 (DMP1), Bone Sialoprotein (BSP) and inflammatory cytokines tumor necrosis factor-α (TNF-α), interleukin-1β (IL-1β), interleukin-6 (IL-6), interleukin-8 (IL-8) were evaluated by using qPCR. Ribonucleic acid (RNA) was extracted from each group of DPCs by using TRIzol (Invitrogen); the total RNA was then reverse-transcribed to cDNA by using the Moloney murine leukemia virus reverse transcriptase (Promega, Madison, WI, USA). qPCR was performed by using SYBR Green Master Mix (Roche, Indianapolis, IN, USA) with 0.5 μL cDNA and 200 nM specific primers (listed in Table 1); the housekeeping gene GAPDH served as an internal control. The reactions were performed by using an ABI PRISM 7500 Sequence Detection System (Applied Biosystems, Foster City, CA, USA). The results were analyzed by using Prism 6 software. 

### 2.11. Enzyme-Linked Immunosorbent Assay (ELISA)

DPCs were seeded at a density of 1 × 105 cells/well in a 6-well plate and were then treated with LPS. Cells treated with the membrane extract were cultured for 7 days. Cell supernatants were collected; the levels of inflammatory cytokines TNF-α, IL-1β, and IL-6 were measured using enzyme-linked immunosorbent assay kits (MM-0181H2, MM-0049H2, and MM-0122H2; Enzyme Immunobiology). The absorbance was measured at 450 nm by using a microplate reader. The concentrations of the cytokines in the cells were analyzed by using Prism 6.0 software.

### 2.12. Western Blot

DPCs were cultured for 14 days in osteogenic media with the membrane extract for Western blot assays. The cells were harvested with a protein lysis buffer (Applygen, Beijing, China). The protein concentration was determined by a BCA protein analysis kit (Pierce, Rockford, IL, USA). Equal aliquots of 40 µg of total protein were separated by sodium dodecyl sulfate-polyacrylamide gel electrophoresis (SDS–PAGE) and transferred to polyvinylidene difluoride membranes (PVDF) (Millipore, Bedford, MA, USA). After blocking in 5% nonfat dry milk for 1 h, the proteins of interest were probed with primary antibodies overnight at 4 °C: DSPP, DMP1, and osteocalcin (OCN) (Santa Cruz Biotechnology, Santa Cruz, CA, USA). After being incubated with secondary antibodies for 1 h, the immunoblots were detected by the Western enhanced chemiluminescence blotting kit (ECL, Applygen, Beijing, China).

### 2.13. Pulp Capping in the Rat Model

Healthy upper first molars from Sprague Dawley rats were used to establish the experimental pulpitis model for this investigation. Rats were anesthetized by using intraperitoneal injections of 10% chloral hydrate (Hushi, Shanghai, China). Cavity preparation and pulp chamber opening were performed by using high-speed turbine tooth drill burs (BR49, ISO001008) with a terminal diameter of 0.5 mm. Coronal pulpal tissue was damaged by using sterile #40 K-files, treated for 30 min with sterile cotton balls that had been soaked in 1 μg/mL of LPS, and then rinse with saline; bleeding was controlled with a small cotton pellet with gentle pressure. The ASP/PLGA-ASP/ACP/PLLA-PLGA composite membrane was placed in the cavity over the exposed pulp. Mineral trioxide aggregate (MTA; Dentsply, York, PA, USA) was used to treat the contralateral cavity as a control. The cavity was then sealed with glass-ionomer cement (GIC; Fuji IX, Shizuoka, Japan). After 8 weeks in a specific pathogen-free laboratory, the rats were sacrificed by CO_2_ inhalation. The specimens were then fixed, decalcified, and sectioned (4 μm). Hematoxylin–eosin staining was performed, and DMP1 expression was detected via immunohistochemistry.

### 2.14. Statistical Analysis

Statistical analyses were performed by using SPSS Statistics 23.0 software (IBM Corp., Armonk, NY, USA). The groups were compared by using a one-way analysis of variance. *p*-values < 0.05 were considered statistically significant. 

## 3. Results

### 3.1. Characterization of Electrospinning Composite Membranes

**Figure 1:**
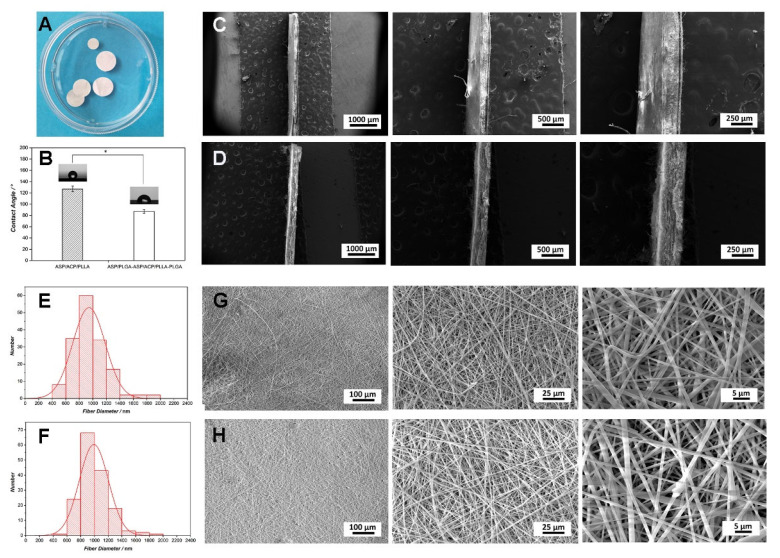
The surface and cross-section morphology, diameter distribution, and hydrophilicity of ASP/PLGA-ASP/ACP/PLLA-PLGA membranes. (**A**) The morphology of ASP/PLGA-ASP/ACP/PLLA-PLGA membranes under the camera. (**B**) The contact angle of the ASP/PLGA-ASP/ACP/PLLA-PLGA membrane was significantly lower than that of the ASP/ACP/PLLA membrane. (**C**–**H**) Diameter distribution and surface as well as cross-section microscopic images of electrospun membranes. (**C**,**E**,**G**) ASP/PLGA-ASP/ACP/PLLA-PLGA membrane; (**D**,**F**,**H**) ASP/ACP/PLLA membrane. * *p* < 0.05.

**Figure 2:**
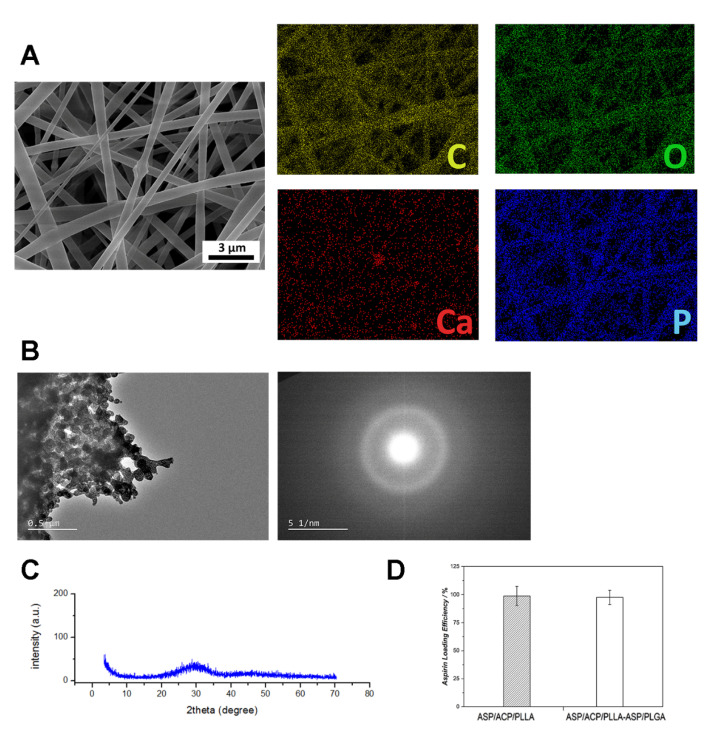
The element composition of ASP/PLGA-ASP/ACP/PLLA-PLGA membranes and the crystalline phases of ACP. (**A**) C, O, Ca, and P element distribution of the ASP/ACP/PLLA membrane. (**B**) TEM images and (**C**) XRD pattern of the ACP sample. (**D**) The aspirin loading efficiency of the ASP/ACP/PLLA and ASP/PLGA-ASP/ACP/PLLA-PLGA membranes.

Figure 1A shows the morphology of ASP/PLGA-ASP/ACP/PLLA-PLGA membranes under a camera. Membranes were observed with white and smooth surface features. SEM was further used to determine the morphological details of the membranes. It can be seen in Figure 1E–H that the electrospun membranes were composed of many nanofibers, the diameters of which mainly ranged from 600 nm to 1400 nm. Although the nanofibers of the ASP/PLGA-ASP/ACP/PLLA-PLGA and ASP/ACP/PLLA membranes exhibited no difference at low magnifications, there are some protuberances presenting on the PLLA membrane at higher magnifications. EDS mapping (Figure 2A) was employed here to analyze the chemical composition of these protuberances. The results revealed strong Ca and P signals at the protuberance site, indicating these protuberances were the agglomerated ACP powders that we added in. To determine that the ACP powders were in amorphous phases, TEM and XRD were carried out here. Figure 2B reveals the TEM morphology of ACP samples. The samples showed a curvilinear appearance with a typical diffraction pattern of an amorphous halo ring. Furthermore, the XRD pattern (Figure 2C) showed no discernable peaks of crystalline calcium phosphate but a characteristic hump of an amorphous phase at around 30°, which was consistent with the TEM results. Apart from the difference in the nanofibers, there were also some differences in the cross-section structure between the ASP/ACP/PLLA and ASP/PLGA-ASP/ACP/PLLA-PLGA membranes. The cross-section images of the ASP/PLGA-ASP/ACP/PLLA-PLGA membrane (Figure 1C) clearly exhibited a sandwich structure composed of three layers of membranes, while the ASP/ACP/PLLA membrane was only observed with a single layer of the membrane (Figure 1D). The contact angle of ASP/ACP/PLLA (inner layer) and PLGA (outer layer) was tested to find out the hydrophilic change in the membrane before and after PLGA coverage. Figure 1B shows that the ASP/PLGA-ASP/ACP/PLLA-PLGA membrane possessed a smaller contact angle (87.1 ± 3.4°) as compared to that of the ASP/ACP/PLLA membrane (126.8 ± 5.1°), showing the improvement effect of PLGA coating on the hydrophilicity of the ASP/ACP/PLLA membrane.

### 3.2. In Vitro Degradation and Release Experiments

**Figure 3:**
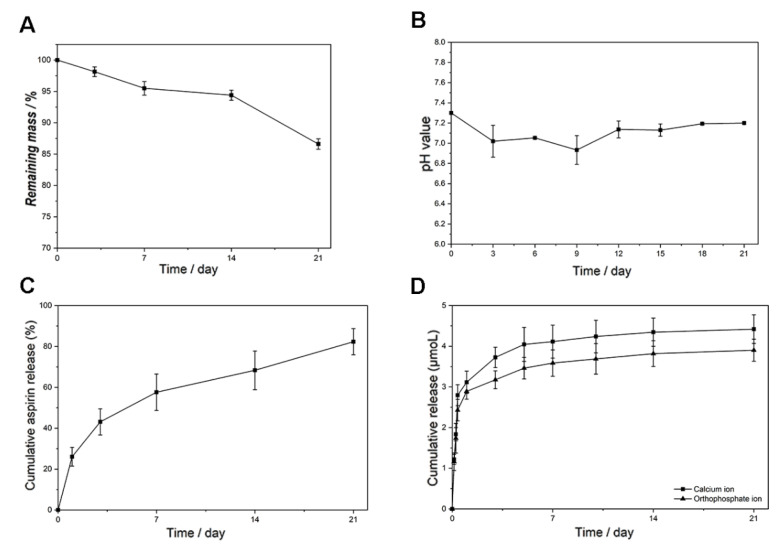
The degradation and release properties of ASP/PLGA-ASP/ACP/PLLA-PLGA membranes. (**A**) The remaining mass, (**B**) media pH value, (**C**) aspirin release profile, and (**D**) ion release profiles over a period of 21 days.

The in vitro degradation experiment was conducted by measuring the mass loss and media pH variation over a period of 21 days. The mass loss of the electrospun membrane stayed at a slow but constant speed during the whole in vitro experiment. It can be seen from Figure 3A that the remaining mass of the membrane was 85% of the original mass at the end of the study. Figure 3B shows that the pH value of the degradation media was lower in the first nine days while being higher the rest of the time. During the whole degradation experiment, the pH value varied in a relatively small range: from 6.8 to 7.2. As for the in vitro release experiments, both the aspirin and ion release profiles were tested. Figure 2D shows that almost all of the aspirin was successfully loaded in the ASP/ACP/PLLA and ASP/PLGA-ASP/ACP/PLLA-PLGA membranes. The aspirin release profile of the ASP/PLGA-ASP/ACP/PLLA-PLGA membrane is shown in Figure 3C. It can be seen that the aspirin release rate was fast in the first week; more than half of the loading amount was released during this time. After day seven, a decreased release rate was observed, and almost 80% of the aspirin was released from the membrane at the end of the experiment. Figure 3D exhibits the release curves of Ca^2+^ and PO_4_^3−^. Compared with aspirin, the ion release rates were somewhat faster, with the observation of an obvious initial burst release. Approximately 75% of the Ca^2+^ and PO_4_^3−^ were released from the membrane on the first day. After the burst release, the Ca^2+^ and PO_4_^3−^ were released in a slower but constant pattern until day 21.

### 3.3. Effects of the Composite Membrane on DPCs Proliferation, Adhesion and Migration

**Figure 4:**
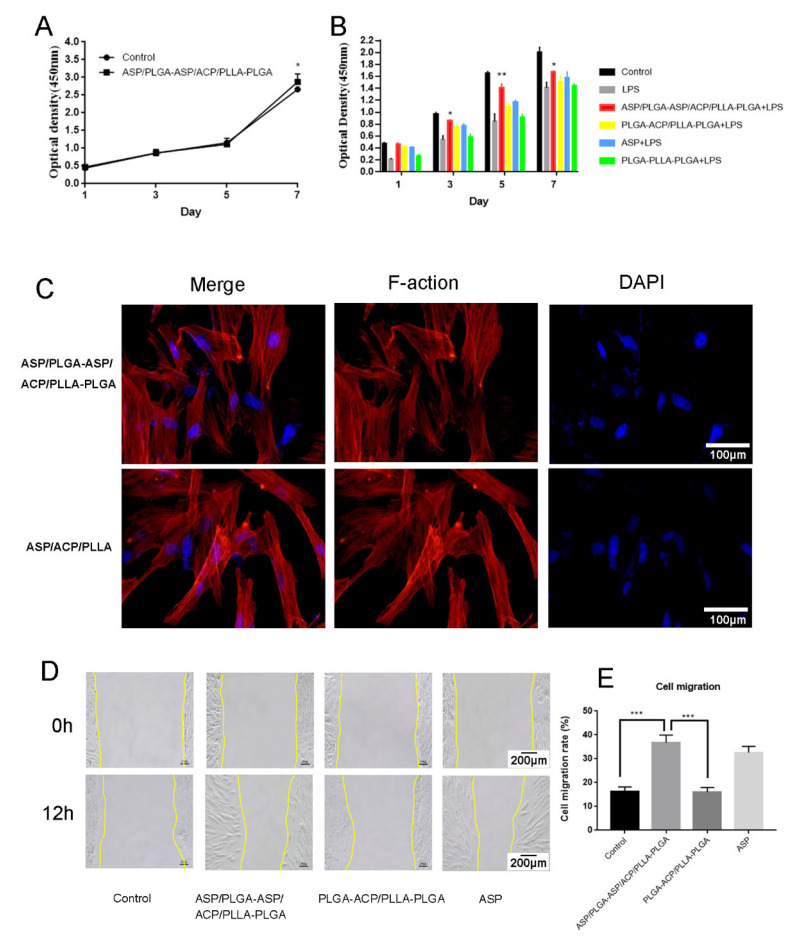
Effects of the ASP/PLGA-ASP/ACP/PLLA-PLGA composite membrane on DPCs proliferation, adhesion, and migration. (**A**) A CCK-8 assay showed stable DPCs growth. There was a significant difference in the cell proliferation rate between the ASP/PLGA-ASP/ACP/PLLA-PLGA and the control group on day 7 (p < 0.05). (**B**) A CCK8 assay showed the effects of the ASP/PLGA-ASP/ACP/PLLA-PLGA composite membrane on DPCs proliferation after LPS treatment. (**C**) Adhered DAPI-labeled DPCs on the ASP/PLGA-ASP/ACP/PLLA-PLGA and ASP/ACP/PLLA membranes were visualized by using a confocal microscope at 24 h. (**D**,**E**) Cell migration rates in the ASP/PLGA-ASP/ACP/PLLA-PLGA and other groups. (* *p* < 0.05, ** *p* < 0.01, and *** *p* < 0.001).

The CCK-8 analysis showed that the DPCs treated with the composite membrane extract grew stably, with proliferation rates higher than the control group on day seven (Figure 4A). DPCs proliferation rate significantly decreased after LPS treatment. The proliferation rate increased when ASP was added; it further increased after ASP/PLGA-ASP/ACP/PLLA-PLGA treatment. There were significant differences among groups in terms of the numbers of viable cells on 1, 5, and 7 days. (p < 0.05) (Figure 4B). DPCs were inoculated on the composite membrane surface and stained with phalloidin/DAPI; Figure 4C indicates that DPCs could colonize and grow on the composite membrane. The cell scratch test was used to examine cell migration abilities; the DPCs migration rate was significantly higher after treatment with ASP and the composite membrane compared with other groups (Figure 4D,E).

### 3.4. Effects of Composite Membrane on the Mineralization and Odontogenic Differentiation of iDPCs

**Figure 5:**
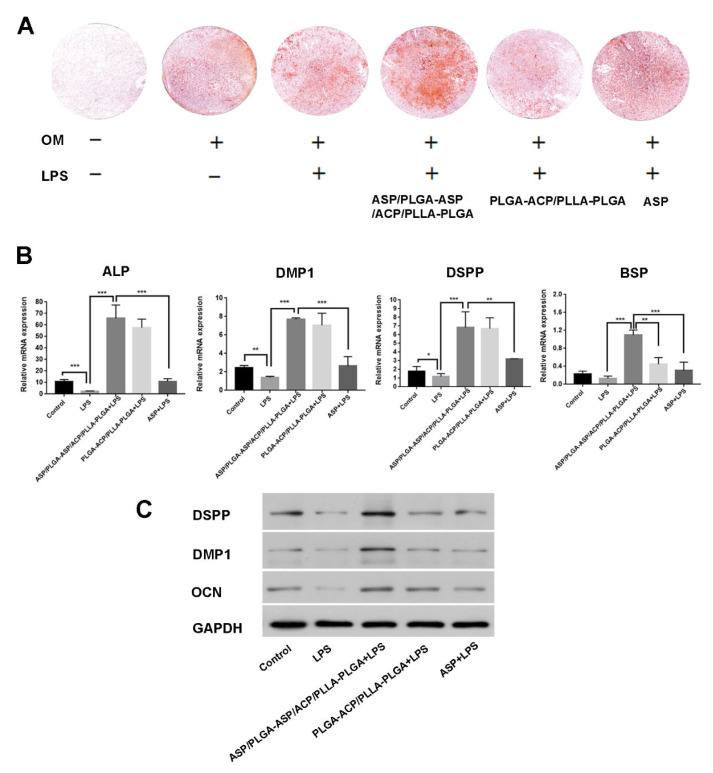
Effects of the ASP/PLGA-ASP/ACP/PLLA-PLGA composite membrane on mineralization and odontogenesis expression in DPCs. (**A**) Mineralized nodule formation was detected by using alizarin red staining on day 21. (**B**) mRNA expression levels of ALP, DMP1, DSPP, and BSP were detected by using qPCR on day 14. (**C**) The protein expression levels of DSPP, DMP1, and OCN by using Western blot on day 14. (* *p* < 0.05, ** *p* < 0.01, and *** *p* < 0.001).

The mineralization-inducing ability of the composite membrane was assessed by using ARS. After 21 days of culturing in osteogenic media, mineralized nodules were stained red with ARS. Mineralized nodule formation significantly decreased after LPS treatment, while it increased after the addition of ASP and PLGA-ACP/PLLA-PLGA. The ASP/PLGA-ASP/ACP/PLLA-PLGA group demonstrated significantly more mineralization according to the ARS findings (Figure 5A). The odontogenic differentiation ability was investigated via qPCR and Western blot through the detection of differentiation-related biomarkers. Both RNA expression levels of ALP, DMP1, DSPP, BSP and protein expression levels of DMP1, DSPP, OCN were reduced in dental pulp cells under inflammatory conditions. they recovered or increased after ASP or PLGA-ACP/PLLA-PLGA treatment and significantly increased after ASP/PLGA-ASP/ACP/PLLA-PLGA membrane treatment. (Figure 5B,C). 

### 3.5. Effects of the Composite Membrane on Inflammatory Cytokine Expression in iDPCs

**Figure 6:**
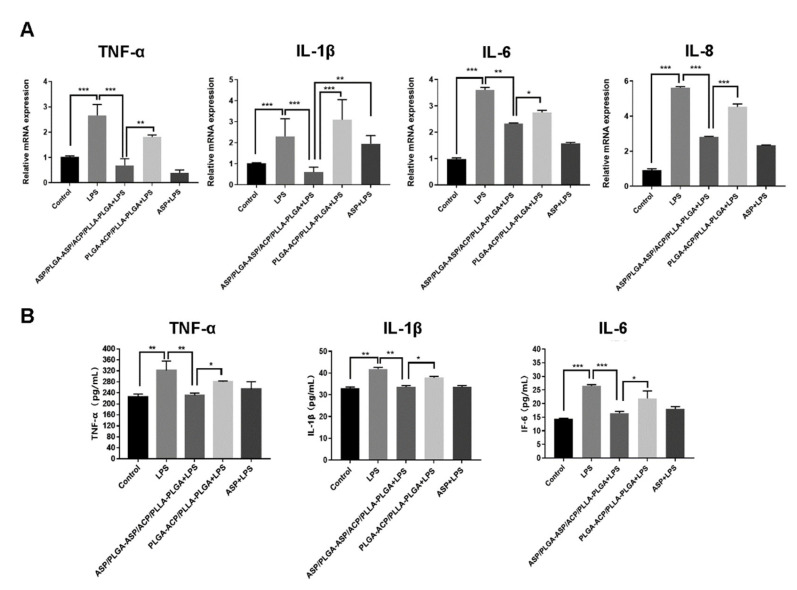
Effects of the ASP/PLGA-ASP/ACP/PLLA-PLGA composite membrane on cytokine expression in DPCs. (**A**) The mRNA expression levels of IL-1β, IL-6, IL-8, and TNF-α were detected by a qPCR analysis on day 7. (**B**) The IL-1β, IL-6, and TNF-α expression levels in the cell supernatant were determined by using enzyme-linked immunosorbent assays. (* *p* < 0.05, ** *p* < 0.01, and *** *p* < 0.001).

In order to assess the anti-inflammatory effects of the composite membrane, the levels of inflammatory biomarkers were measured by using qPCR. The mRNA expression levels of the inflammatory cytokines IL-1β, IL-6, TNF-α, and IL-8 were higher in iDPCs than in DPCs. After treatment with ASP or the ASP/PLGA-ASP/ACP/PLLA-PLGA composite membrane, the mRNA expression levels of inflammatory cytokines significantly decreased in iDPCs (Figure 6A). Enzyme-linked immunosorbent assays also showed that ASP and composite membrane treatments significantly downregulated the expression levels of TNF-α, IL-1β, and IL-6 (Figure 6B).

### 3.6. Effect of the ASP/PLGA-ASP/ACP/PLLA-PLGA Composite Membrane for Dental-Pulp-Capping In Vivo 

**Figure 7:**
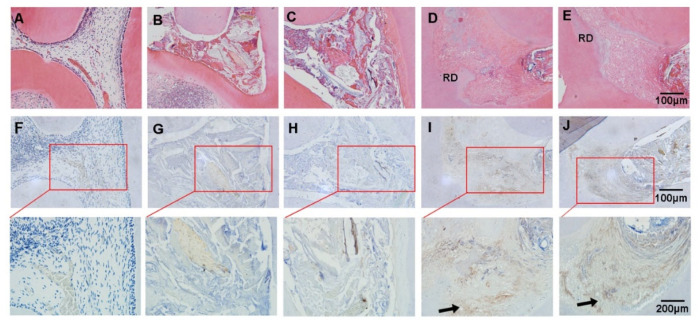
Hematoxylin–eosin staining (**A**–**E**) and immunohistochemical staining (**F**–**J**) showed newly formed tissues. (**A**,**F**) Normal group, (**B**,**G**) LPS group, (**C**,**H**) LPS + GIC group, (**D**,**I**) MTA + GIC group, and (**E**,**J**) ASP/PLGA-ASP/ACP/PLLA-PLGA + GIC group. RD: reparative dentin-like tissue. The arrows indicate DMP1-positive cells.

The in vivo effects of the ASP/PLGA-ASP/ACP/PLLA-PLGA composite membrane as a pulp-capping material was investigated in a rat dental pulpitis model. Pulp tissues treated with LPS became necrotic at eight weeks (Figure 7B,C). Newly formed dentin-like tissue was observed in both the ASP/PLGA-ASP/ACP/PLLA-PLGA (Figure 7D) and MTA groups (Figure 7E) at eight weeks. The immunohistochemical staining results showed higher DMP1 expression in the ASP/PLGA-ASP/ACP/PLLA-PLGA group than in the MTA group (Figure 7I,J).

## 4. Discussion

Dental caries and trauma often lead to dental pulp exposure. The dysplasia of enamel and dentin caused by some genetic diseases may also lead to dental pulp tissue being vulnerable to damage [21,22,23]. Vital pulp therapy is important for clinical treatment because vital pulp tissue promotes root development in young permanent teeth [24,25]; it also provides resistance to biting forces, thus reducing the risk of root fractures [26,27]. The long-term prognosis of teeth treated by root canal therapy is worse than vital pulp teeth [28]. The biological basis for vital pulp therapy lies in the repair and regeneration capacity of the dental pulp. The dental pulp contains many undifferentiated mesenchymal stem cells with high proliferation and multi-differentiation potentials [29]. When the pulp is infected, odontoblasts become seriously damaged, and various inflammatory cytokines are produced [30]. Under these circumstances, prompt infection control allows DPCs to migrate to the injury site and repair the defective dentin, thus forming a hard tissue barrier and enabling the re-establishment of tissue homeostasis and health [31,32]. Conversely, persistent inflammation induces an immune response that can lead to tissue necrosis. Therefore, the timely control of early pulpitis is crucial for dental pulp repair and regeneration [33,34,35].

The anti-inflammatory drug, ASP, used in this study exerts favorable anti-inflammatory effects by inhibiting cell cyclooxygenase, blocking the conversion of prostaglandins into arachidonic acid, and reducing the production of inflammatory mediators. It can also inhibit the expression of nuclear-factor-κB-mediated inflammatory cytokines (e.g., TNF-α), which has important anti-inflammatory effects [36,37,38,39]. Aspirin is widely used in bone regeneration [40]. Studies have confirmed that aspirin can promote the osteogenic differentiation of mesenchymal stem cells and inhibits the expression of inflammatory factors to activate osteoblasts and inhibit osteoclasts [41]. The effect of aspirin on bone regeneration is dose-dependent. The intervention of low-dose aspirin on mesenchymal cells may inhibit the differentiation and formation of RANKL-induced osteoclasts as well as significantly reduce the number of trap-positive osteoclasts [42]. The dose of aspirin is an important factor affecting cell proliferation, differentiation, and migration. High doses of aspirin will inhibit cell proliferation [43]. In recent years, many studies have loaded aspirin on different materials, and the composite application of these materials is conducive to controlling the released concentration of aspirin. Similarly, the effect of aspirin on dental pulp cells is dose-dependent [44]. Low concentrations of ASP can promote DPCs proliferation and reduce the expression levels of inflammatory cytokines, including TNF-α and IL-6. Low concentrations of ASP can also promote odontoblast differentiation of DPCs through the Wnt/β-catenin pathway [44,45,46]. However, there is some evidence that high concentrations of ASP can inhibit DPCs proliferation and lead to cell death [47]. Therefore, an optimal ASP concentration is necessary for effective inflammation control and the avoidance of cell death during pulp capping. In this study, the lower layer of the composite membrane quickly released high concentrations of ASP to inhibit the early stages of inflammation; the middle layer allowed for the long-term slow release of low concentrations of ASP during the later stages of inflammation, thus promoting DPCs proliferation and differentiation. When studying the effect of anti-inflammatory drugs on cells in an inflammatory state, in vitro inflammatory models are usually used. LPS is often used to establish an inflammatory microenvironment in vitro [48,49,50]. According to previous studies, treating human dental pulp cells (HDPCs) with LPS with a concentration of 1 µg/mL is the appropriate method to simulate an inflammatory microenvironment in vitro [51]. Therefore, this study used 1 μg/mL LPS to establish an in vitro inflammatory microenvironment model of HDPCs for in vitro research. Our results showed that the expression levels of the inflammatory cytokines IL-1β, IL-6, IL-8, and TNF-α were significantly increased in LPS-induced inflammatory-reacted dental pulp cells, but they decreased after treatment with the composite membrane. Thus, ASP has a positive effect on the repair of the inflammatory dental pulp.

Ideally, pulp capping should be able to repair the damaged pulp and the material can be replaced by reparative dentin. Therefore, the pulp-capping material should be biodegradable. Meanwhile, some physical and chemical properties of pulp-capping materials, such as the pH value and calcium ion release, are also crucial because they will have a direct impact on the repair process of dental pulp tissue [52,53]. ACP is a hydroxyapatite precursor with good biocompatibility and biodegradability [54,55]. It can alter cell function and tissue differentiation by releasing calcium ions to promote cell osteogenic differentiation [56]. ACP/PLLA scaffolds can continuously release calcium and phosphorus ions. The biological activity of a pulp-capping agent is related to its ability to release calcium ions. Studies have confirmed that increasing the concentration of calcium ions can increase the expression of osteocalcin (OPN) and bone morphogenetic protein-2 (BMP-2) as well as stimulate the release of proteoglycans and growth factors of a mineralized dentin matrix. Then, the undifferentiated pulp stem cells migrate to the injury site, proliferate, and differentiate to form an extracellular matrix and promote mineralization [52,57]. The ACP-mediated directional differentiation of mesenchymal stem cells is affected by the concentration of free calcium ions; if the concentration decreases in the surrounding environment, osteogenic differentiation is reduced [58]. As a drug delivery carrier and scaffold material, PLLA has some problems, such as its weak stability of hydrophilic components and the burst release phenomenon, which need to be improved [59]. For the slow and long-term release of calcium ions, our ACP/PLLA scaffolds were coated with the PLGA membrane, which is biocompatible and has a sustained release capacity. This membrane can regulate the degradation rate, ensure the long-term maintenance of the drug concentration, and avoid “peak and valley” phenomena [60,61]. In this study, the release of calcium and phosphorus ions from the composite membrane was steady, suggesting that the structure of the composite membrane could achieve the controlled release of calcium and phosphorus ions. The metabolites of PLLA in the body are lactic acid, and the metabolites of PLGA in the body are lactic acid and glycolic acid. These monomers are metabolized in the body through the tricarboxylic acid cycle, and the final metabolites, carbon dioxide and water, have no toxic effect on cells [62,63,64,65]. PLGA and PLLA can be used as suitable microcarriers to achieve sustained drug release [66,67]. As a scaffold material, PLGA is conducive to the colonization of cells (such as SHED, DPSCs, and dental pulp fibroblasts), and on PLGA scaffolds, stem cells can differentiate into odontoblast-like cells and endothelial cells, producing tissues similar to dental pulp and dentin [68]. The outer PLGA membrane layer improves the superficial hydrophilicity of the composite membrane [69,70]. This hydrophilic property ameliorates the biocompatibility of the ASP/PLGA-ASP/ACP/PLLA-PLGA membrane, as well as increasing its favorability for cell adhesion [71,72]. The rapid adhesion of cells will initiate the differentiation process in the early stage, resulting in a satisfactory odontogenic differentiation at the end [73,74,75]. Moreover, an acidic environment not only suppresses the viability of bone-related cells but also inhibits their mineralization process [76,77]. Previous studies have confirmed that an increase in alkaline pH will enhance the expression of BMP-2 mRNA and ALP activity in dental pulp cells, but that exorbitant pH will lead to excessive calcium ion release, induce dystrophic calcification in exposed dental pulp areas, reduce the volume of the reparative dental pulp, and may hinder any future dental pulp treatment [57,78]. In this work, the addition of ACP, an alkaline agent [79], in the membrane neutralizes the acidic degradation byproducts of PLLA and PLGA in addition to maintaining a neutral environment, which we believe is also conducive to odontogenic differentiation.

In this study, the effect of the composite membrane on iDPCs proliferation and odontoblastic differentiation after LPS treatment was more than the effects of ASP or ACP/PLLA/PLGA scaffolds alone. This finding is potentially the case because the early release of ASP controlled inflammation and created a suitable environment for cell proliferation and differentiation, while the subsequent controlled release of ASP and ACP promoted a microenvironment that aided in pulp tissue repair.

The purpose of this paper was to solve the problem of preserving vital pulp in clinical practice. The rat pulp-capping model was used for eight weeks of observations. In future studies, the observation time should be extended, and large animals should be used to further observe the pulp-capping effect of the material. In addition, for the release of anti-inflammatory drugs, such as aspirin, in pulp-capping materials, inflammatory responsive controlled release materials can be further designed for different inflammatory states so as to more accurately regulate the pulp inflammation state. This paper provides a new idea and theoretical basis for the design and application of vital dental pulp preservation materials.

## 5. Conclusions

In this study, the composite application of aspirin, amorphous calcium phosphate, and PLLA/PLGA scaffold materials was used to develop a pulp-capping material with multiple effects for the repair of the injured dental pulp. The ASP/PLGA-ASP/ACP/PLLA-PLGA composite membrane developed in this study allowed for the sequential release of the anti-inflammatory drug aspirin as well as the calcium/phosphorus ions required for mineralization; The mass loss of the membrane stayed at a slow but constant speed, with a relatively stable pH value during degradation; it also promoted the proliferation and odontogenic differentiation of iDPCs, while inhibiting inflammatory cytokine release. It had the dual effects of reducing inflammation and promoting mineralized barrier formation. The ASP/PLGA-ASP/ACP/PLLA-PLGA composite membrane represents a promising vital pulp conservation material with the potential for clinical applications.

The application of composite materials for pulp therapy can combine the advantages of multiple materials, but it also faces great challenges because the mechanism of action between composites is complex and needs to be further explored. We will further evaluate the biological properties of the material and conduct long-term in vivo studies to assess the effectiveness of ASP/PLGA-ASP/ACP/PLLA-PLGA composite membrane as a vital pulp-capping agent in clinical applications.

## Figures and Tables

**Table 1:** Primers used for quantitative PCR.

Target Gene	Sequence	
GAPDH	forward:	GAGAAGGCTGGGGCTCATTT
	reverse:	TAAGCAGTTGGTGGTGCAGG
ALP	forward:	ATCTTCCTGGGCGATGGGAT
	reverse:	CCACATATGGGAAGCGGTCC
DMP1	forward:	TTGTGAACTACGGAGGGTAGAGG
	reverse:	CTGCTCTCCAAGGGTGGTG
DSPP	forward:	CATGGGCCATTCCAGTTCCTC
	reverse:	TCATGCACCAGGACACCACT
BSP	forward:	CGATTTCCAGTTCAGGGCAGT
	reverse:	TCCATAGCCCAGTGTTGTAGC
TNF-α	forward:	CACTTTGGAGTGATCGGCCC
	reverse:	CAGCTTGAGGGTTTGCTACAAC
IL-1β	forward:	TTCGAGGCACAAGGCACAA
	reverse:	TGGCTGCTTCAGACACTTGAG
IL-6	forward:	CATCCTCGACGGCATCTCAG
	reverse:	TCACCAGGCAAGTCTCCTCA
IL-8	forward:	AGTTTTTGAAGAGGGCTGAGA
	reverse:	TGCTTGAAGTTTCACTGGCATC

## Data Availability

The data presented in this study are available on request from the corresponding author.

## Abbreviations

BSP	Bone Sialoprotein
DCM	Dichloromethane
DMF	Dimethyl Formamide
DMP	Dentin matrix protein
DPCs	Dental pulp stem cells
DSPP	Dentin sialophosphoprotein
EDS	Energy-dispersive spectrometer
GIC	Glass-ionomer cement
HDPCs	Human dental pulp cells
iDPCs	Inflamed dental pulp stem cells
IL	Interleukin
LPS	Lipopolysaccharide
MTA	Mineral trioxide aggregate
OCN	Osteocalcin
OPN	Osteopontin
PBS	Phosphate buffered solution
PLGA	Poly (lactic-co-glycolic acid)
PLLA	Poly (L-lactic acid)
qPCR	Quantitative Polymerase Chain Reaction
RNA	Ribonucleic Acid
SEM	Scanning electron microscope
SHED	Stem cells from human exfoliated deciduous teeth
TEM	Transmission electron microscope
TNF	Tumor necrosis factor
XRD	X-ray diffraction
α-MEM	Minimum Essential Medium-Alpha
